# Removal of Stains and Spots

**Published:** 1881-06

**Authors:** 


### REMOVAL OF STAINS AND SPOTS.

*Stearine.*—In all cases, strong, pure alcohol.

*Gum, Sugar, Jelly, Etc.*—Simple washing with water at a hand heat.

*Matter Adhering Mechanically.*—Beating, brushing, and currents of water either on the upper or under side.

*Lime and Alkalies.*—White goods, simple washing. Colored cottons, woolens and silks are moistened, and very dilute citric acid is applied with the finger end.

*Alizarine Inks.*—White goods, tartaric acid, the more concentrated the older the spots. On colored cottons and woolens, and on silks, dilute tartaric acid is applied cautiously.

*Scorching.*—White goods, rub well with linen rags dipped in chlorine water; colored cottons, re-dye if possible, or in woolens raise a fresh surface; silks, no remedy.

*Oil Colors, Varnish and Resins.*—On white or colored linens, cottons or woolens, use rectified oil of turpentine, alcohol, lye and then soap; on silks, use benzine, ether, and mild soap, very cautiously.

*Vegetable Colors, Fruit, Red Wine and Red Ink.*—On white goods, sulphur fumes or chlorine water; colored cottons and woolens, wash with lukewarm soap, lye, or ammonia; silk, the same, but more cautiously.

*Iron Spots and Black Ink.*—White goods, hot oxalic acid, dilute muriatic acid, with little fragments of tin. On fast-dyed cottons and woolens, citric acid is cautiously and repeatedly applied; silks, impossible.

*Blood and Albumenoid Matters.*—Steeping in lukewarm water. If pepsine, or the juice of *Carica papaya*, can be procured, the spots are first softened with lukewarm water, and then either of these substances is applied.

*Grease.*—White goods, wash with soap or alkaline lyes; colored cottons, wash with lukewarm soap lyes; colored woolens, the same, or ammonia; silks, absorb with French chalk or Fuller's earth, and dissolve away with benzine or ether.

*Tanning from Chestnuts, Green Walnuts, etc., or Leather.*—White goods, hot chlorine water and concentrated tartaric acid; colored cottons, woolens and silks, apply dilute chlorine water cautiously to the spot, washing it away and reapplying it several times.

*Tar, Cart-Wheel Grease, Mixtures of Fat, Resin, Carbon and*

*Acetic Acid.*—On white goods, soap and oil of turpentine, alternating with streams of water; colored cottons or woolens, rub in with lard and let lie, soap and let lie again, and treat alternately with oil of turpentine and water; silks, the same, but more carefully, using benzine instead of oil of turpentine.

*Acids, Vinegar, Sour Wine, Must and Sour Fruits.*—White goods, simple washing, followed up by chlorine water. Colored cottons, woolens and silks are very carefully moistened with dilute ammonia with the finger end. In case of delicate colors, it will be found preferable to make some prepared chalk into a thin paste with water, and apply it to the spots.—*Chicago Journal of Science.*

### FALLING IN LOVE.

The days of romance are not past. A Cuban planter visiting New York saw a charming woman on a Brooklyn ferryboat and fell in love with her. He traced her to her home and learned that she was a widow most respectably connected. He was called to Cuba, and wrote her a letter full of affection and giving references. Her friends inquired and found that he was a desirable match. She replied to the letter. He responded. She wrote and he wrote until there was an offer of marriage and an acceptance, and the wedding day was fixed. She prepared her bridal robe and he returned to New York. They met at one of her friend's, he anticipating a second vision of beauty. She saw a handsome man; he looked and screamed, "You are the wrong woman!" And so it was. They had neglected to exchange photographs. She remains a widow, and he haunts the Brooklyn ferryboats for another vision.

### PHOTOGRAPHIC MUSIC.

An English paper tells of a gentleman, who, on being asked to sing, produced from his pocket a little case, which contained his music, photographed down to the size of note paper. He had duplicate copies of each song, and handed one to the accompanist, singing from the other himself. The expedient saved all the bother of bringing a roll of music, unfolding it, collecting it again, and so forth.

TEACHER: "Suppose that you have two sticks of candy, and your big brother gives you two more, how many have you got then?" Little boy (shaking his head): "You don't know him; he ain't that kind of a boy."

### A PATENT AND PATTERN MOTHER.

A physician of superior abilities in a neighboring state had a daughter of sweet seventeen, whose health was not as good as was desired, and not wishing to give her medicine, he made arrangements for having her travel leisurely through New England for several months, as the most likely means of her restoration. At the same time her mother obtained a supply of various patent medicines, and placed them in the travelling trunk, *with private instructions* to take them while travelling. The daughter to accommodate herself and her father, took the journey, and in obedience to the mother, took the physic, and as a matter of course, is—still an invalid. The father remains in ignorance of the cause of the failure of beneficial results from his prescription.

Lesson.—*The child who privately does a thing for one parent, known to be contrary to the wishes of the other, and the parent who can counsel it, are equally guilty of a violence against domestic rule, which will not cease to bear the pernicious fruits of deception and discord, to the latest hour of their lives.*

